# A Novel Endothelial Damage Inhibitor Reduces Oxidative Stress and Improves Cellular Integrity in Radial Artery Grafts for Coronary Artery Bypass

**DOI:** 10.3389/fcvm.2021.736503

**Published:** 2021-10-06

**Authors:** Thomas Aschacher, Ulrike Baranyi, Olivia Aschacher, Eva Eichmair, Barbara Messner, Daniel Zimpfer, Roxana Moayedifar, Guenther Laufer, Maximilian Y. Emmert, Sigrid E. Sandner

**Affiliations:** ^1^Department of Cardio-Vascular Surgery, Clinic Floridsdorf and Karl Landsteiner Institute for Cardio-Vascular Research, Vienna, Austria; ^2^Department of Cardiac Surgery, Medical University Vienna, Vienna, Austria; ^3^Department of Plastic, Reconstructive and Plastic Surgery, Medical University Vienna, Vienna, Austria; ^4^Cardiovascular Surgery, Charite-Universitätsmedizin Berlin, Berlin, Germany; ^5^Department of Cardiothoracic and Vascular Surgery, German Heart Institute Berlin, Berlin, Germany

**Keywords:** endothelial damage inhibitor, radial artery, bypass graft failure, coronary artery bypass grafting, oxidative damage

## Abstract

The radial artery (RA) is a frequently used conduit in coronary artery bypass grafting (CABG). Endothelial injury incurred during graft harvesting promotes oxidative damage, which leads to graft disease and graft failure. We evaluated the protective effect of DuraGraft®, an endothelial damage inhibitor (EDI), on RA grafts. We further compared the protective effect of the EDI between RA grafts and saphenous vein grafts (SVG). Samples of RA (*n* = 10) and SVG (*n* = 13) from 23 patients undergoing CABG were flushed and preserved with either EDI or heparinized Ringer's lactate solution (RL). The effect of EDI vs. RL on endothelial damage was evaluated *ex vivo* and *in vitro* using histological analysis, immunofluorescence staining, Western blot, and scanning electron microscopy. EDI-treated RA grafts showed a significant reduction of endothelial and sub-endothelial damage. Lower level of reactive oxygen species (ROS) after EDI treatment was correlated with a reduction of hypoxic damage (eNOS and Caveolin-1) and significant increase of oxidation-reduction potential. Additionally, an increased expression of TGFβ, PDGFα/β, and HO-1 which are indicative for vascular protective function were observed after EDI exposure. EDI treatment preserves functionality and integrity of endothelial and intimal cells. Therefore, EDI may have the potential to reduce the occurrence of graft disease and failure in RA grafts in patients undergoing CABG.

## Introduction

Coronary artery bypass grafts experience stress resulting from the physical trauma of harvesting and handling, post-harvest ischemia, oxidative stress, reperfusion injury and adaptive stress in the new post-grafting environment (1, 2). This may lead to thrombosis, intimal hyperplasia (IH) and atherosclerosis, and ultimately to bypass graft failure (2). Loss of nitric oxide (NO) is an early event causing about 5 to 15% of grafts to occlude within the first month of coronary artery bypass grafting (CABG) (1, 3, 4). NO is produced in the blood vessel wall mainly by eNOS and may be scavenged by excess reactive oxygen species (ROS). Uncontrolled ROS production leads to impaired endothelial function and consequently to vascular dysfunction.

Appropriate intraoperative preservation of free grafts is important to maintain graft viability and patency (5–8). This is a particular issue in saphenous vein grafts (SVG) but may also be relevant in other free grafts such as the radial artery (RA) (9–11). Recent studies have suggested a protective effect of DuraGraft®, an endothelial damage inhibitor (EDI), for one-time intraoperative graft treatment during CABG (10, 12, 13).

While previous studies have reported the effect of EDI in the context of SVG, only little is known about its effect on the RA.

Substantial observational evidence indicates that multiple arterial grafting is associated with improved patient outcomes (14). Recently, a patient-level pooled analysis of randomized trials comparing the RA and SVG reported a lower risk of a composite of cardiovascular outcomes including mortality at 10 years with the RA (15). The 2018 EACTS/ESC Guidelines on myocardial revascularization support use of the RA with a Class I, level of evidence A recommendation (16).

Here, we assessed the protective effect of the EDI DuraGraft on RA in patients undergoing CABG. We analyzed whether the EDI decreases the adverse effects of hypoxic conditions, remodels the endothelial barrier and preserves the immune-mediated regeneration capacity of endothelial cells (EC).

## Materials and Methods

### Patients and Tissue Sample Collection

Vascular tissue samples were collected intraoperatively from 23 patients undergoing routine isolated CABG procedures (RA, *n* = 10; SVG, *n* = 13). Vein harvesting was done by endoscopically technique, radial artery harvesting by open technique. One graft segment per patient, approximately 2 cm in length, were obtained and divided. One half was treated immediately with the EDI according to manufacturer's instructions, and the other half with heparinized lactated Ringer's solution (RL). The samples were not tested by pressurized injections. Samples were preserved in the respective solutions for 60 min at room temperature and transferred to the laboratory for further processing.

The study was approved by the Ethics Committee of the Medical University of Vienna (EC Number: 2061/2018). Written informed consent was obtained from all patients prior to inclusion in the study. All samples were rendered anonymous by using ID numbers for data analysis and laboratory protocols. The data underlying this article will be shared upon reasonable request to the corresponding author. The use of all human cells and tissues was approved by the institutional ethics review board and complies with the Declaration of Helsinki. Patients with recent (<1 year) or current administration of immunomodulatory agents (e.g., thalidomide, lenalidomide), chemotherapeutical or immunosuppressive agents (e.g., cortisone, cytokines), recent (<6 months) RA puncture for cardiac catheterization, concomitant immune disease, positive virology (HIV, hepatitis-A/-B/-C, and influenza), diabetes mellitus on insulin >6 months prior to CABG, were excluded from participation in the study to avoid potential interactions with the effect of the EDI and to reduce inhomogeneity among the study population.

### Preservation Solution and Treatment

In this study we used the EDI DuraGraft® (Somahlution, Jupiter, FL, United States). It is an ionically and pH-balanced physiological salt solution containing glutathione, L-ascorbic acid, and L-arginine and other protective ingredients. The components and proposed mechanism of action of DuraGraft® have been previously described (12). In brief, DuraGraft is an intraoperative treatment solution that protects the structural integrity and function of the vascular endothelium, and protects the conduit from the damaging effects of ischaemia during storage and reperfusion. Heparinized (2 IE Heparin/mL) lactated Ringer's solution (RL) (B. Braun, Austria) served as a control. After harvest, either the EDI or RL was used to carefully flush and store the RA or SVG prior to anastomosis.

### EC and Vascular Smooth Muscle Cells (vSMC) Isolation, Cell Cycle and Cell Cytotoxic Analysis

We analyzed cellular-cytotoxic and cell-growth conditions in EC and vSMC culture treated with EDI or RL. Cell culture medium (EGM^TM^-2 Endothelial Cell Growth Medium-2, Lonza, Slough, UK) was used as additional control. Cell isolation from specimens was performed according to previously described protocol (17). Viability was assessed using an EZ4U kit (Biomedica MP, Vienna, Austria). Cell cycle and annexin V-staining were performed as previously described (18).

### RNA Isolation and Real-Time PCR (RT-qPCR)

RNA isolation, cDNA synthesis and RT-qPCR were performed as described previously (18). Samples were normalized to the geometric mean of reference genes. Oligonucleotide-primer sequences are listed in Supplemental Materials.

### Protein Analysis

Cultured cells and tissue were analyzed by Western blot as described previously (18). Detection was performed with ECL Ultra Western blot HRP Substrate (Lumigen, Inc.) and quantified with ImageQuant TL 7.0 (GE Healthcare, Vienna, Austria).

Immunofluorescence (IF) staining has been described previously (18). DNA were counterstained with DAPI for cell nuclei visualization and equally cell analysis, as well internal fluorescence control (ThermoFisher Scientific, Vienna, Austria). Microscopy pictures were taken on a Confocal Laser Scanning Microscope 700 (Zeiss, Vienna, Austria). Images were analyzed with the CellProfiler™ cell image analysis software. For histochemical staining formalin-fixed tissue samples were processed routinely and sections stained by Elastica van Gieson for histopathologic examination. All antibodies used are listed in Supplemental Materials.

### Scanning Electron Microscopy (SEM)

RA and SVG segments were mounted onto cryo-SEM stubs with 25% dextran and plunged into liquid nitrogen. Gold sputter coated samples were viewed with a Hitachi S4700 Field Emission SEM using an accelerating voltage of 2 kV and a working distance of 6.5 mm.

### Luciferase Assay

The activity of the HO-1 promotor regulatory region was measured as previously described (19). All experiments were performed at least three times for each plasmid, and the average relative luciferase activity is reported. The used sequences were: Wild- TGGAAGGCCTTCTTTCTAGAGAGGGAATT; Mutant- TGGAAGGCCTTCTTAGAAGAGAGGGAATT.

### Evaluation of Hypoxic Induced Cell Response/Damage, and Oxidation-Reduction Potential

The hypoxic induced ROS increase was analyzed by redox analyser (Redox Sys, Aytu Bioscience, US) following manufacturer's protocol.

### ROS/RNS Measurements by OxySelect Assay

Baseline ROS/RNS levels of treated grafts were analyzed by OxiSelect^TM^ *In vitro* ROS/RNS Assay Kit according to the manufacturer's protocol (Cell Biolabs, Inc., San Diego, CA). A H_2_O_2_ standard curve was prepared for each plate and statistical analysis.

### Statistical Analysis

Statistical analysis was performed with GraphPad Prism Version 6 (GraphPad Software, CA, USA) and SPSS 15.0 software (SPSS Inc, Chicago, USA). Comparisons between groups were performed by two-tailed Student's *t*-test or analysis of variance (ANOVA). Comparisons between multiple groups were tested by Multi-Way ANOVA. Variables were evaluated by Spearman's rank correlation. *P* < 0.05 was considered statistically significant.

## Results

### Patient Characteristics

RA and SVG samples were obtained from patients undergoing CABG. Patient baseline characteristics are presented in Supplementary Table 1. Baseline levels of ROS/RNS of graft protein lysates assayed using OxySelect^TM^ method correlated significantly with presence of diabetes and with age in RA, and with age in SVG, and level of eNOS correlated significantly with age (Supplementary Tables 2, 3).

### Structural Changes, Regeneration Ability and Cellular Damage

**Figure 1:**
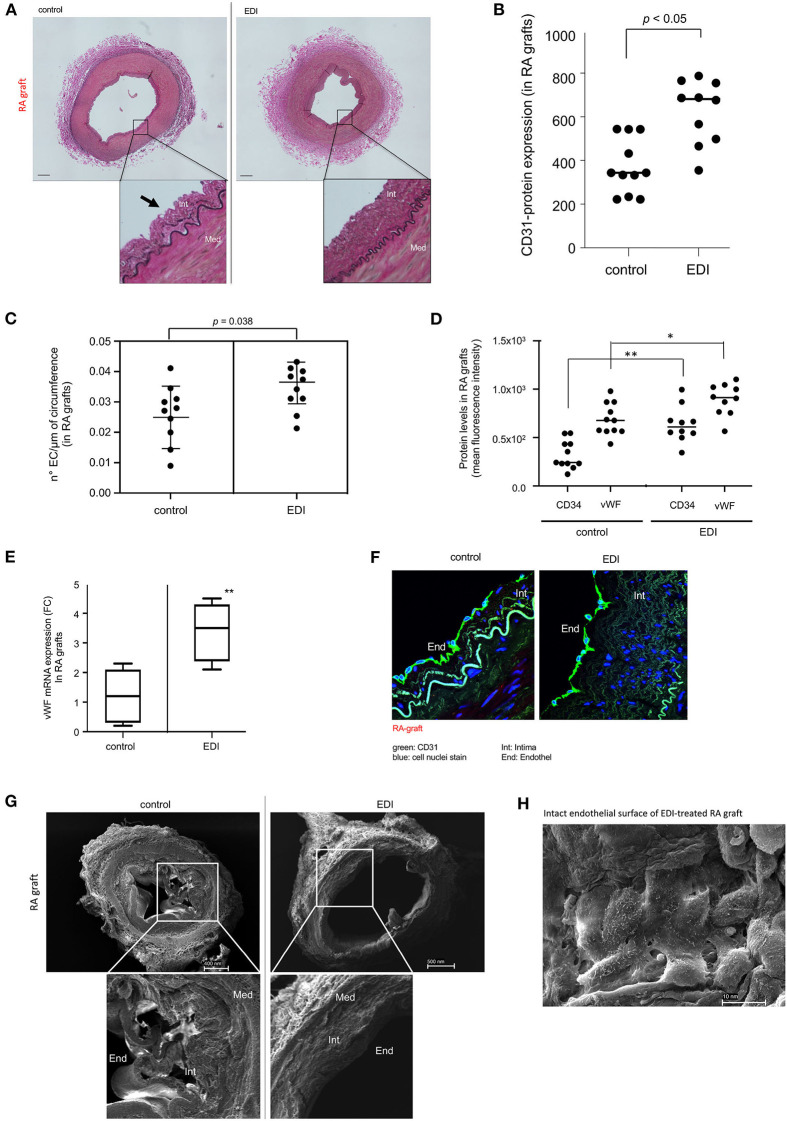
Endothelial damage inhibitor (EDI) maintained homeostasis and integrity of endothelial cells (EC). **(A)** Photomicrographs of RA grafts treated with RL or EDI. RL treatment shows flattened intimal EC (black arrow). scale bar, 70μm. **(B)** Quantitative analysis of CD31 IF staining in RA sections. **(C)** Quantitative analysis of EC count lining in the circumference of RA. SD is given. **(D)** Significant decrease of CD34 and vWF in RL-treated RA grafts. *p < 0.05; **p < 0.01. **(E)** vWF mRNA expression by qRT-PCR assay in EDI vs. RL **p < 0.01. **(F)** Representative image of CD31 IF staining in EDI-treated RA grafts. scale bar: 20μm; green, CD31; blue, cell nuclei staining (DAPI). **(G)** Representative SEM image of EDI and RL treated RA grafts. EC, endothelial cells; Int, intima; Med, media. **(H)** Top view on cross section of EDI vs. RL-treated RA samples with intact endothelial layer. scale bar, 10 nm.

We assessed whether the EDI reduces endothelial cell injury and intimal swelling to examine its effect on endothelial integrity. Histological analysis showed partial endothelial denudation, and an irregular structure of sub-endothelial and intimal layer in control compared with EDI-treated RA grafts (Figure 1A). The adventitial layer showed incipient detachment from the medial layer in RL-treated segments. We measured level of endothelial marker CD31 to confirm endothelial detachment. Partial endothelial denudation appeared responsible for the significant loss of CD31 in control group, which was also associated with a significant reduction of ECs compared to EDI-treated RA grafts (Figures 1B,C). CD34 and von Willebrand factor (vWF) were significantly overexpressed in EDI-treated RA grafts (Figure 1D). Correspondingly, gene expression analysis demonstrated significantly retained high expression of vWF mRNA after EDI treatment (Figure 1E).

The specific morphological structure of subendothelial and intimal layer between EDI-treated and RL-treated RA grafts were visualized by CD31 IF staining and SEM (Figures 1F,G). After RL treatment the endothelial layer showed erratic cellular structure and beginning of detachment from intima and the intimal layer compared to EDI treatment, which showed a balanced compact structure (Figure 1G). Detailed SEM images showed an intact cell-to-cell connection between ECs after EDI treatment (Figure 1H).

We found similar cell protective effects of EDI on endothelial and subendothelial layer integrity in SVG (Supplementary Figures 1, 2).

In summary, the endothelial and sub-endothelial layer was preserved in both types of bypass grafts after EDI treatment, while RL-treated grafts showed damaged endothelial surface and beginning incongruence of intimal structure.

### Proliferation and Viability of EC After EDI Exposure

**Figure 2:**
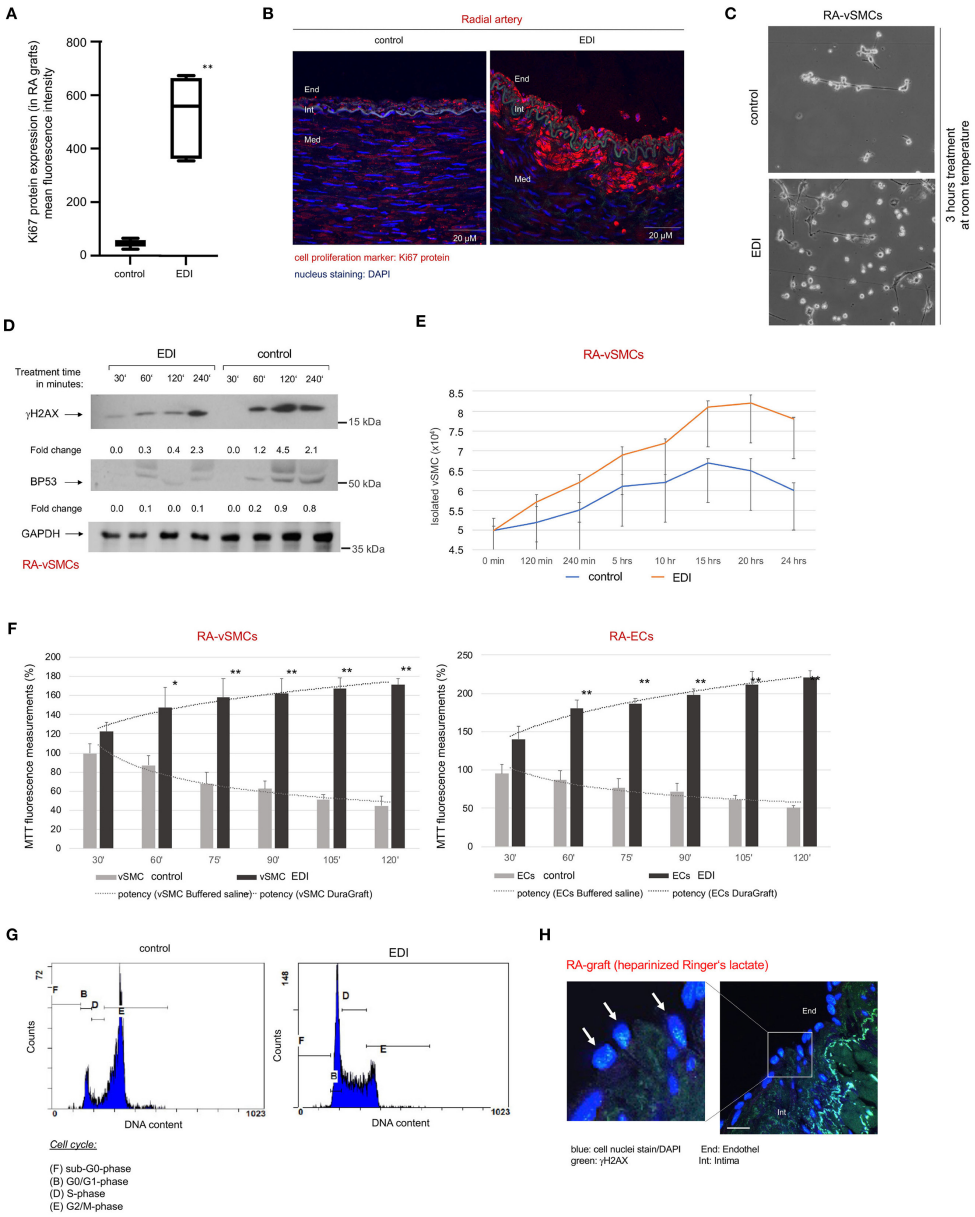
Increased cellular metabolism together with reduced cellular senescence, DNA damage and cell death in EDI-treated ECs, vSMC and conduit tissue. **(A)** Statistical analysis and **(B)** Representative image of Ki67 IF staining in RA grafts after treatment with RL or EDI. **p < 0.01; End, endothel; Int, intima; Med, media. scale bar, 20μm. **(C–G)** ECs and vSMCs were isolated (RA-ECs, RA-vSMCs) followed by treatment with cell specific cell culture medium, EDI or RL in a time-dependent manner. **(C)** Representative light microscopy image of RA-vSMCs cell culture cultivation 3 hours after treatment start. **(D)** *p*53 and γH2AX marker stained in RA samples after exposure to EDI or RL in a time dependent manner. GAPDH was used as internal control. Fold change was calculated to 30′ time point. **(E)** Reduction of cell growth was measured by cell counting in control group. **(F)** Cell metabolic activity was measured by MTT assay. *p < 0.05; **p < 0.01. **(G)** Cell cycle analysis indicated congruent results for cell senescence (G2/M phase) in control group by flow cytometry. **(H)** Representative image of RL-treated samples measured by IF staining. blue, cell nuclei (DAPI); green, γH2AX. scale bar, 10 μm. vSMC, vascular smooth muscle cells; EC, endothelial cells.

We analyzed the viability of ECs and vSMCs of bypass grafts. Expression of the cell proliferation marker Ki67 was significantly reduced in RL-treated RA (Figures 2A,B) compared to retained proliferation capacity of EDI-treated RA.

We observed reduced cell growth in cultivated vSMCs of RA grafts treated with RL when compared to EDI solution (Figure 2C). This reduced cell proliferation may be a manifestation of hypoxic DNA damage, cell senescence and cell death (20). Therefore, cell senescence and DNA damage were assessed by γH2AX staining. We found a significant increase of γH2AX-expression with RL treatment, together with an increase of apoptosis marker *p*53-expression (Figure 2D). In contrast, in EDI-treated graft segments suggested a protective effect against DNA damage and cell senescence. A maintained proliferation rate of EDI treated RA-vSMCs was confirmed by cell proliferation assay (Figure 2E) and metabolism assay. Both showed a significant protective effect of EDI treatment on RA-vSMCs and ECs isolated from RA grafts (Figure 2F). Analysis of the cell cycle by flow cytometry was in line with these results (Figure 2G). IF staining revealed a significant increase of γH2AX positive signals in EC at the endothelial layer of RL-treated RA (Figure 2H). The same beneficial results were observed for EDI-treated SVG (Supplementary Figure 3).

In summary, our analyses showed a reduction of proliferation and increased cell senescence in RL-treated RA grafts and SVG, which may be considered a response to intracellular stress by hypoxic conditions after harvesting.

### EDI Treatment Reduces Hypoxic Damage

**Figure 3:**
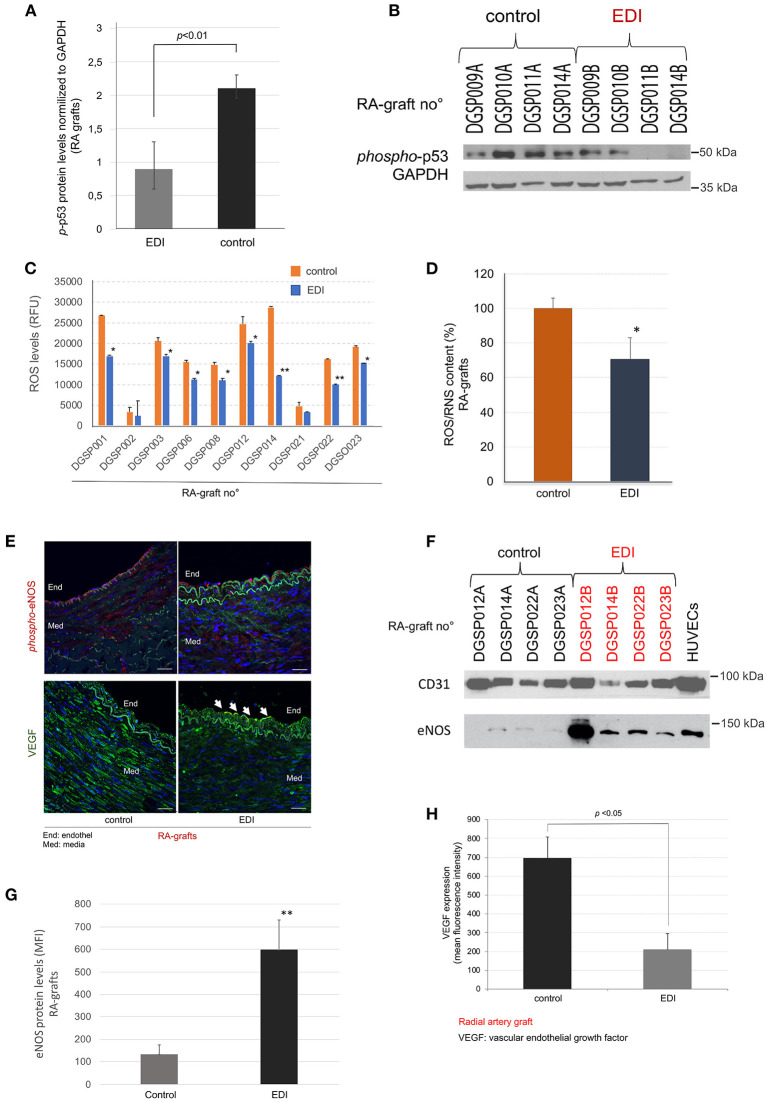
EDI treatment reduced ROS/RNS mediated cell defense. *p*-p53 mediated induction of cell apoptosis measured by Western blot (WB). **(A)** Statistic calculation of *p-p*53 normalized to internal control GAPDH after EDI or RL treatment. **(B)** Representative WB is shown. RA graft samples (RA graft no°) are given on the top, the last letter indicates treatment (A = RL, B = EDI). (C+D) ROS/RNS measurement by OxiSelect™ in protein extracts of RA samples. **(C)** The blot shows RA graft no° specific results. *p < 0.05; **p < 0.01. **(D)** ROS/RNS content in RA samples calculated relative to control group. *p < 0.05. **(E)** Representative image of phosphorylated-eNOS (*phospho-*eNOS, red) and VEGF (green) protein expression stained by IF. Endothelial (End) layer (white arrows). Int, intima. Scale bar, 20μm. **(F)** Representative WB of treated RA samples stained for eNOS and the reference endothelial marker CD31. ECs are given as positive control. The last letter of RA-graft no° indicated the treatment solution (A = RL, B = EDI). (G+H) Statistical analysis for eNOS protein expression **(G)**, and VEGF protein expression **(H)** measured by IF are given.

We measured cell death induced by hypoxic damage in RA grafts. The phosphorylated *p*53 (*p*-*p*53) protein was used as a marker of apoptosis. While EDI-treated RA grafts showed normal levels of cell apoptosis signals, RL treatment leads to a significant increase of *p*-p53 levels (Figures 3A,B). SVG showed similar favorable effects after EDI treatment (Supplementary Figures 3, 4).

OxySelect^TM^ measurement of ROS/RNS indicated a significant increase of hypoxic induced ROS/RNS production in the control group, whereas EDI treatment had a protective inhibitory effect (Figures 3C,D). Further, while SVG and RA samples showed a reduction of ROS production, RA grafts presented a more pronounced outcome (Supplementary Figure 5).

Next, the non-activated eNOS and activated (phosphorylated) eNOS (*p*-eNOS) expression, which impairs endothelial function (21, 22), was analyzed in correlation with ROS/RNS levels. IF staining demonstrated a significantly higher expression of *p*-eNOS in the endothelial layer, but not in the subendothelial layer of the RL-treated RA grafts (Figure 3E), while eNOS protein showed significantly elevated levels in EDI-treated RA grafts in all layers (Figures 3F,G).

For endothelial specific protein expression, we analyzed the ratio of eNOS to the endothelial marker CD31. Our findings indicated activation during hypoxic stress in these sections. In accordance with hypoxic stress induced rapid intimal swelling, we measured VEGF, a cellular signal for activated vascular cell proliferation (23). VEGF expression was significantly higher in RL-treated when compared to EDI-treated RA sections (Figure 3H).

In addition, we analyzed eNOS and VEGF protein expression of EDI vs. RL treated SVG, and then compared it to EDI treatment in RA grafts. While *p*-eNOS was not detected, we observed high VEGF expression measured by IF in SVG (Supplementary Figure 6).

Taken together, RL-treated samples appeared to trigger a cascade of *p-*eNOS and VEGF expression after hypoxic stress conditions while hypoxic cell damage was inhibited by EDI in the RA graft sections. Our findings demonstrate improved graft preservation and therefore advantage of RA grafts over SVG.

### Hypoxic-Preventive Effect of EDI on Endothelial NO Synthase Expression and Regeneration Ability of EC

**Figure 4:**
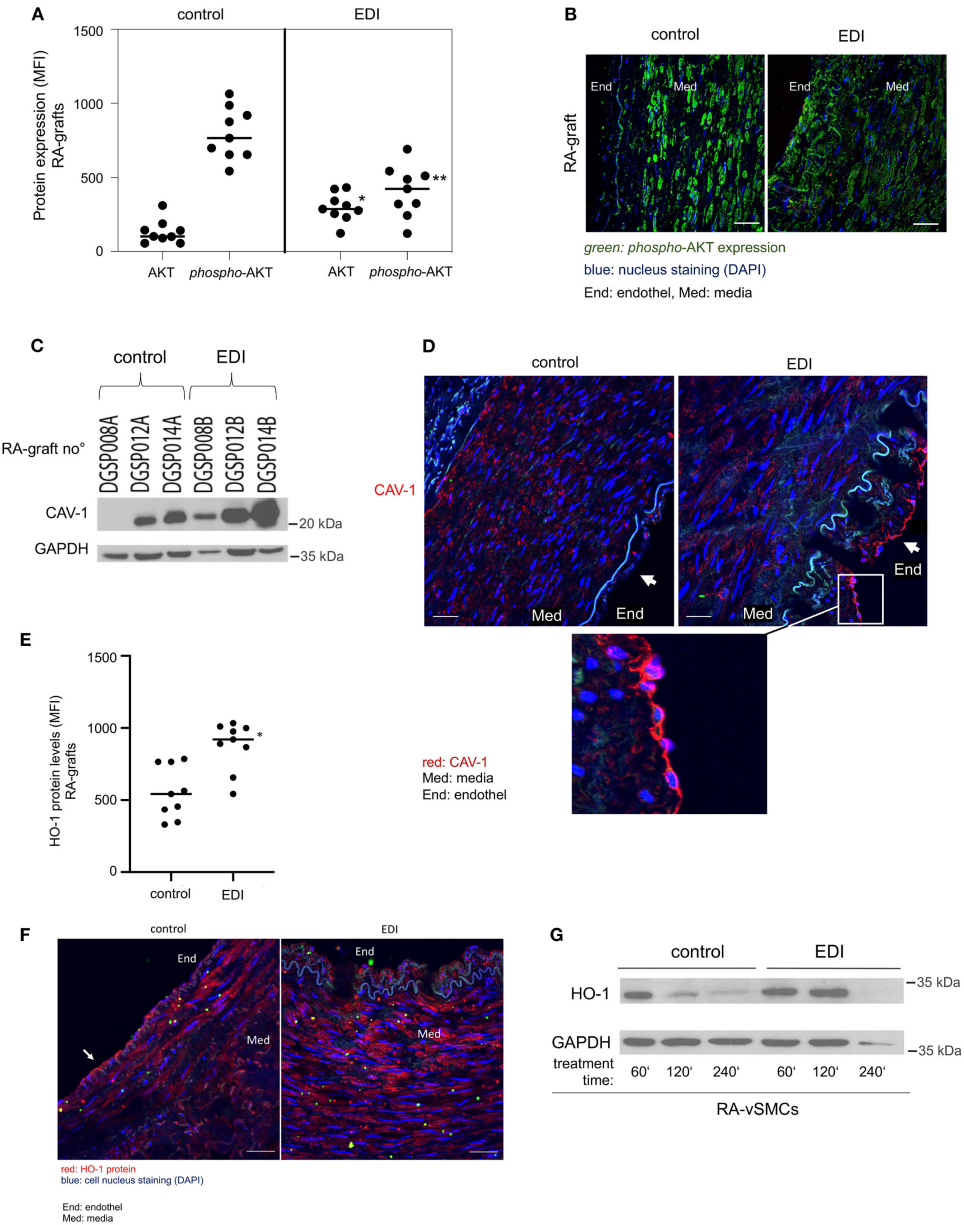
Mediated increased capacity, Caveolin-1 (CAV-1) and heme oxygenase-1 (HO-1) by EDI treatment of vascular tissues. EDI or RL (control) treated patients' specimens were stained for *phosphorylated-*AKT(*p*-AKT) and AKT protein expression. Analysis done by immunofluorescence (IF) staining. **(A)** Statistical calculations and **(B)** representative images were given. **p < 0.01. MFI, mean fluorescence intensity. green, *p*-AKT; blue, nuclei staining (DAPI). End, endothelium; Med, media. (*C*+*D*) CAV-1 protein expression measured by Western blot (WB) and IF analysis. **(C)** Representative CAV-1 WB of RA graft protein extracts (RA graft no°) after EDI treatment compared to control treatment. GAPDH protein was used as internal control. The last letter of RA-graft no° indicated treatment (A=control, B = EDI solution). **(D)** CAV-1 IF staining after EDI treatment compared to control group. red, CAV-1; blue, cell nuclei staining (DAPI); Med, media; End, endothel. **(E–G)** HO-1 expression after EDI or RL exposure in RA specimens of the same patient. **(E)** Statistical analysis of HO-1 protein expression measured by IF staining. *p < 0.05. **(F)** A representative IF image is given. End, endothel; MED, media. red, HO-1; scale bar, 20 μm. **(G)** RA-vSMC were treated with EDI vs. RL. Treatments were done in a time dependent manner.

To detect the *p*-eNOS involved pathway, we analyzed total-AKT and activated phosphorylated AKT (*p*-AKT-Ser^473^) with protein expression experiments. The results demonstrated that the role of ROS in the induction of eNOS is dependent on AKT/*p-*AKT regulation at the transcriptional level in RA grafts (Figures 4A,B). The results showed significant differences of total-AKT and *p-*AKT expression in EDI-treated grafts (Figure 4A). Total-AKT was significantly depleted and active *p*-AKT protein was significantly elevated in RL-treated RA grafts. The elevation in p-AKT in control RA grafts was particularly striking given the reduced levels of total-AKT. Comparison of SVG treated with EDI vs. RL showed significantly higher active *p*-AKT protein levels in the RL-treated group (Supplementary Figure 6C).

Caveolin-1 (CAV-1) is expressed in EC, macrophages and vSMC (24), and acts as a negative regulator of its activity and thus a regulator of NO production (25, 26). Hypoxia has an immediate effect on the endothelial barrier, therefore we focused on the endothelial layer. CAV-1 protein levels were elevated after EDI treatment measured by Western blot (Figure 4C). Moreover, we found retained CAV-1 expression in intact EC of EDI-treated RA (Figure 4D).

HO-1 is a protein whose expression is systemically induced in pathophysiological states associated with oxidative stress and vascular injury (27). As expected, significantly increased HO-1 protein levels were found after EDI treatment in RA grafts (Figures 4E–G, white arrow).

In summary, our findings indicate the protective effect of EDI treatment by decreased ROS levels and less AKT activation, which is in accordance with the eNOS/*p*-eNOS results. The activation of eNOS-counterpart CAV-1 and lower HO-1 levels indicate endothelial dysfunction in the RL-treated group, which was absent after EDI treatment, suggesting that the EDI may efficiently inhibit oxidative cell damage.

### EDI Enhances Redox Potential With Positive Effects on Cellular Regeneration

**Figure 5:**
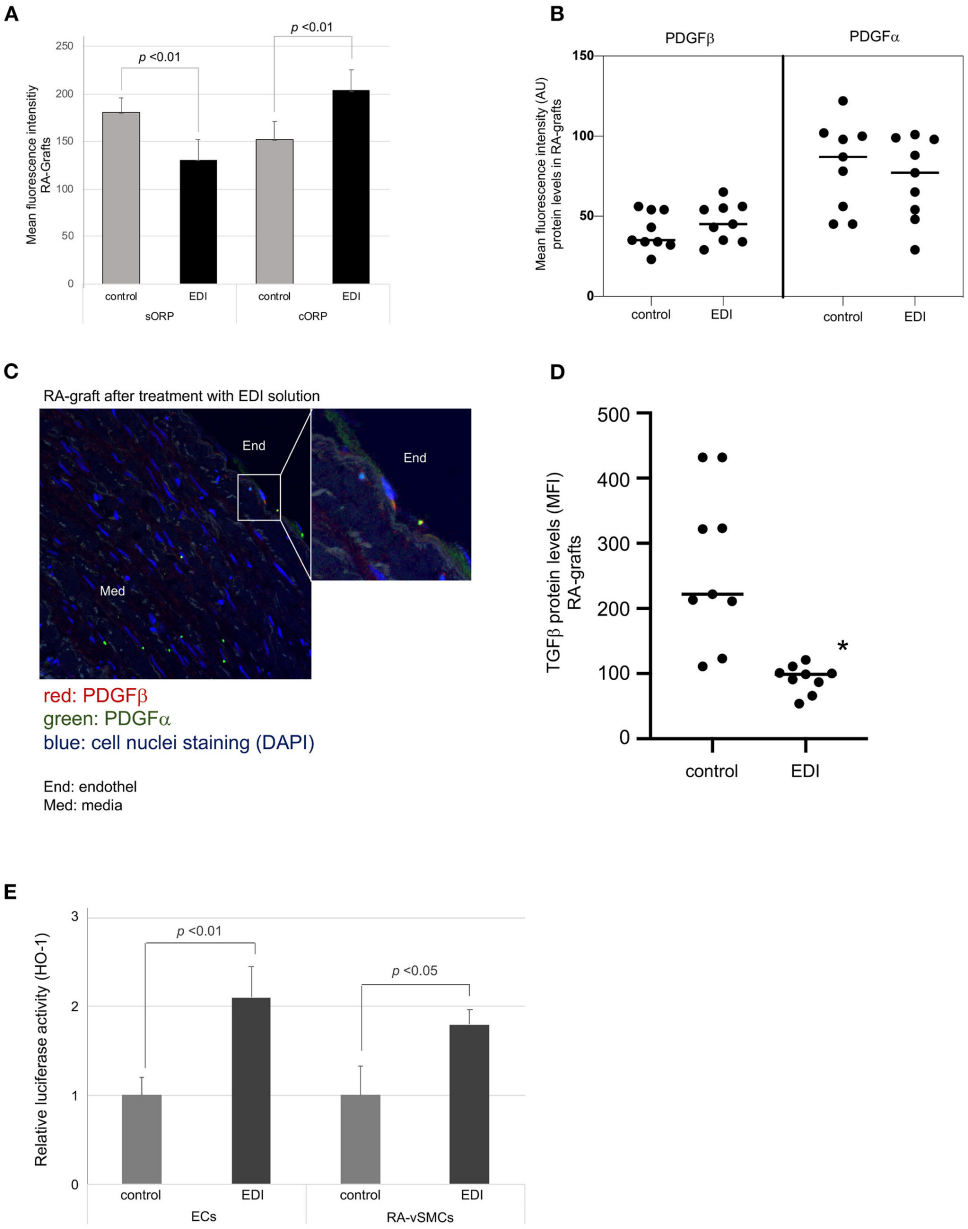
Oxidative damage reduced by EDI treatment. **(A)** RA specimens of the same patients after different treatments (EDI solution vs. control group) were measured for the static oxidation reduction potential (sORP) as well as the capacity ORP (cORP). Protein expression levels of PDGFα and PDGFβ after EDI or RL treatment. **(B)** Dot blot calculation of mean fluorescence intensity (MFI) of both proteins were presented. **(C)** PDGFβ expression of EDI treated RA samples by IF staining. Magnification of endothelial layer is shown. Red, PDGFα; green, PDGFβ; blue, cell nuclei staining (DAPI); End, endothel; Med, media. **(D)** TGFβ expression calculation by MFI indicated protective effect of EDI in RA samples. **(E)** Relative HO-1 promoter activation after EDI or RL exposure measured by luciferase activity assay of heme oxygenase-1 (HO-1) in isolated ECs or vSMC. *P < 0.05.

To test redox potential, we analyzed the static oxidation-reduction potential (sORP) which is proportional to the balance of reductants and oxidants. Low sORP values indicate normal range of oxidative stress, while relative increased values define a higher state of oxidative stress. The capacity of oxidation-reduction potential (cORP) is the measure of antioxidant reserves in the normal range, while lower cORP values describe that the sample is below normal antioxidant reserves (28). sORP and cORP measurements in EDI-treated RA grafts showed significantly higher protective potential against oxidative stress (Figure 5A). The same measurements were performed in SVG samples with a stronger benefit in EDI-treated RA grafts compared to EDI-treated SVG (Supplementary Figure 7).

Platelet-derived growth factor (PDGF) and transforming growth factor-β(TGFβ are important mediators in vascular remodeling, vasculogenesis, and angiogenesis (29). In our study, IF staining showed non-significant changes in PDGFα expression (Figures 5B,C). NO regulates TGFβ signaling in EC (30), and was significantly increased in RL-treated RA grafts (Figure 5D).

An additional important mediator modulating oxidative vascular injury is the induction of HO-1. HO-1 is known to protect against neointimal formation by promoting reendothelialization (31). Therefore, we analyzed mediator induced promotor activation of HO-1 DNA sequence during EDI treatment in our cell culture set by luciferase activity assay. We found a significant increase of promotor activated HO-1 expression in EDI-treated compared to RL-treated ECs and vSMC (Figure 5E). In summary, EDI showed a strong protective ability by activating HO-1 expression, while the RL group demonstrated activation of TGFβ due to oxidative stress and vascular injury.

## Discussion

Recent studies have suggested a favorable effect of EDI on IH and a lower risk of long-term adverse events in patients undergoing CABG with SVG (10, 12, 13, 32). However, for RA grafts evidence on EDI is scarce and its protective effects remain to be elucidated.

The protective effect of EDI is important to maintain the endothelial barrier, while decreasing neointimal formation and cell toxicity (33). Our study demonstrates that EDI preserves the endothelial barrier and intracellular response of RA grafts against hypoxic conditions which may support long-term integrity and function of the conduit. Our results demonstrate that in RA grafts the endothelial-cell specific surface markers CD31, CD34 and vWF are significantly higher and specific morphological features are maintained after EDI treatment when compared to RL treatment.

In an *ex vivo* experiment with ECs and vSMC isolated from patients' specimens, the EDI appears to stabilize extracellular conditions and to preserve cell integrity and cell function of ECs and vSMCs. We further show that EDI reduces hypoxic induced cell senescence and apoptosis, which may prevent excessive overreaction of the immune system and graft environment. Our results demonstrate an increased expression and sensitization of HO-1 and TGFβ downregulation. HO-1 plays a critical role in prevention of vascular inflammation and has important antioxidant and antiapoptotic effects (31). Additionally, we observe a trend of increased PDGFα/β receptors expression localized to EC, which, combined with decreased VEGF expression, may reduce the profibrotic cellular mechanism in the endothelial and intimal layer of graft vessels (34, 35). EDI induced HO-1 expression may improve vascular local effects that converge in the inhibition of proinflammatory activity and regulation of the proliferative response of vSMC, which may prevent graft intimal thickening. However, recent evidence suggests that PKB/AKT may modulate HO-1 activity and its anti-apoptotic effects in presence of cellular stress (36). HO-1 and AKT exert co-dependent cytoprotective effects against oxidative stress-induced apoptosis (37). Our results are suggestive that these two cytoprotective enzyme systems may function cooperatively to inhibit pro-oxidant induced apoptosis of bypass conduits.

We also show that EDI inhibits excessive accumulation of intracellular ROS/RNS and increases redox potential by activating and stabilizing NO production in RA grafts. Both reduced ROS and increased NO are the result of an intact and non-exhausted eNOS and AKT pathway. EDI treatment shows balanced *p*-AKT/AKT and *p*-eNOS/eNOS status, whereas RL treatment shows depleted eNOS protein amount, with insufficient capacity as ROS/RNA counterpart. CAV-1, with regulative feature against overreactive eNOS/AKT activation, is only seen after EDI treatment, suggesting a stable intracellular capacity for balanced hypoxic response, and avoiding related DNA damage. These findings are further verified by specific static and capacity ORP measurements. In addition, our results show significant beneficial effects on relative proportions of oxidants (ROS) to reductants (antioxidants) in EDI treated RA grafts.

NO prevents graft spasm by dilatation of blood vessels and may prevent intimal hyperplasia by inhibition of DNA synthesis, mitogenesis and proliferation of vSMCs (38). eNOS is essential for the function of the endothelial progenitor cells involved in vascular repair and protects smooth muscle from exposure to platelet-derived growth factors (39). However, the EDI used here contains high L-arginine, which is involved in the eNOS cascade by oxidation to L-citrulline and NO (40). Therefore, it may maintain eNOS functionality, while at the same time upregulates the expression of the enzyme. Furthermore, compounds that increase eNOS protein levels are only beneficial when guaranteeing eNOS functionality (41).

However, it is important to recognize as a limitation that the small sample size does not allow any conclusions regarding the impact of clinical characteristics and perioperative medication on the effects of EDI. Further studies with more patients' samples are needed to determine their role.

If and to which extent the beneficial effects observed in our study translate into a clinically meaningful improvement in outcomes after CABG remains to be evaluated in subsequent studies. Results from the ongoing European DuraGraft® Registry that included patients with free arterial grafts treated with DuraGraft® are eagerly awaited to provide longer-term data validating the clinical efficacy of DuraGraft®.

## Conclusion

In summary, during graft harvesting RA conduits are exposed to hypoxia, which leads to ROS production and oxidative cell stress. In the present study we have shown that the EDI solution used is able to maintain extracellular conditions *ex vivo* and prevent DNA damage and cell death. This effect supports maintenance of an intact endothelial barrier, integrity, and function with retained subendothelial cell homeostasis. Intracellular effect of EDI shows decreased ROS production, balanced conditions between oxidative and antioxidant values, and inhibits progressive neointimal formation by downregulation of TGFβ induced VEGF cellular over-proliferation. In addition, we show a direct positive effect on protective markers which are involved in tissue regeneration and cell recovery such as HO-1 and AKT/eNOS pathway. Taken together, EDI treatment of RA grafts may significantly reduce post-grafting re-oxygenation reaction and may have the potential to reduce the occurrence of RA graft disease and failure in patients undergoing CABG.

## Data Availability Statement

The original contributions presented in the study are included in the article/Supplementary Material, further inquiries can be directed to the corresponding author/s.

## Ethics Statement

The study was approved by the Ethics Committee of the Medical University of Vienna (EC Number: 2061/2018). The patients/participants provided their written informed consent to participate in this study.

## Author Contributions

TA: conceptualization, methodology, analysis, project administration, software, visualization, writing—original draft, and writing—review and editing. UB, OA, EE, and BM: methodology, analysis, visualization, and writing—review and editing. DZ, RM, and GL: conceptualization and writing—original draft. ME and SS: conceptualization, supervision, visualization, writing—review and editing, and writing—original draft. All authors contributed to the article and approved the submitted version.

## Conflict of Interest

SS is an investigator in the European VASC registry and a member of the registry scientific advisory committee. ME is the PI of the European VASC registry and the chair of the registry scientific advisory committee. Both have received consulting fees from Somahlution Inc, Jupiter, FL. The remaining authors declare that the research was conducted in the absence of any commercial or financial relationships that could be construed as a potential conflict of interest.

## Publisher's Note

All claims expressed in this article are solely those of the authors and do not necessarily represent those of their affiliated organizations, or those of the publisher, the editors and the reviewers. Any product that may be evaluated in this article, or claim that may be made by its manufacturer, is not guaranteed or endorsed by the publisher.
