# Supplementary material for: A Novel Endothelial Damage Inhibitor Reduces Oxidative Stress and Improves Cellular Integrity in Radial Artery Grafts for Coronary Artery Bypass
Source: Front Cardiovasc Med. 2021 Oct 6;8:736503. doi: 10.3389/fcvm.2021.736503 (PMC8527012; doi:10.3389/fcvm.2021.736503)
Supplement: Supplementary file 1 [file Data_Sheet_1.DOCX]

**
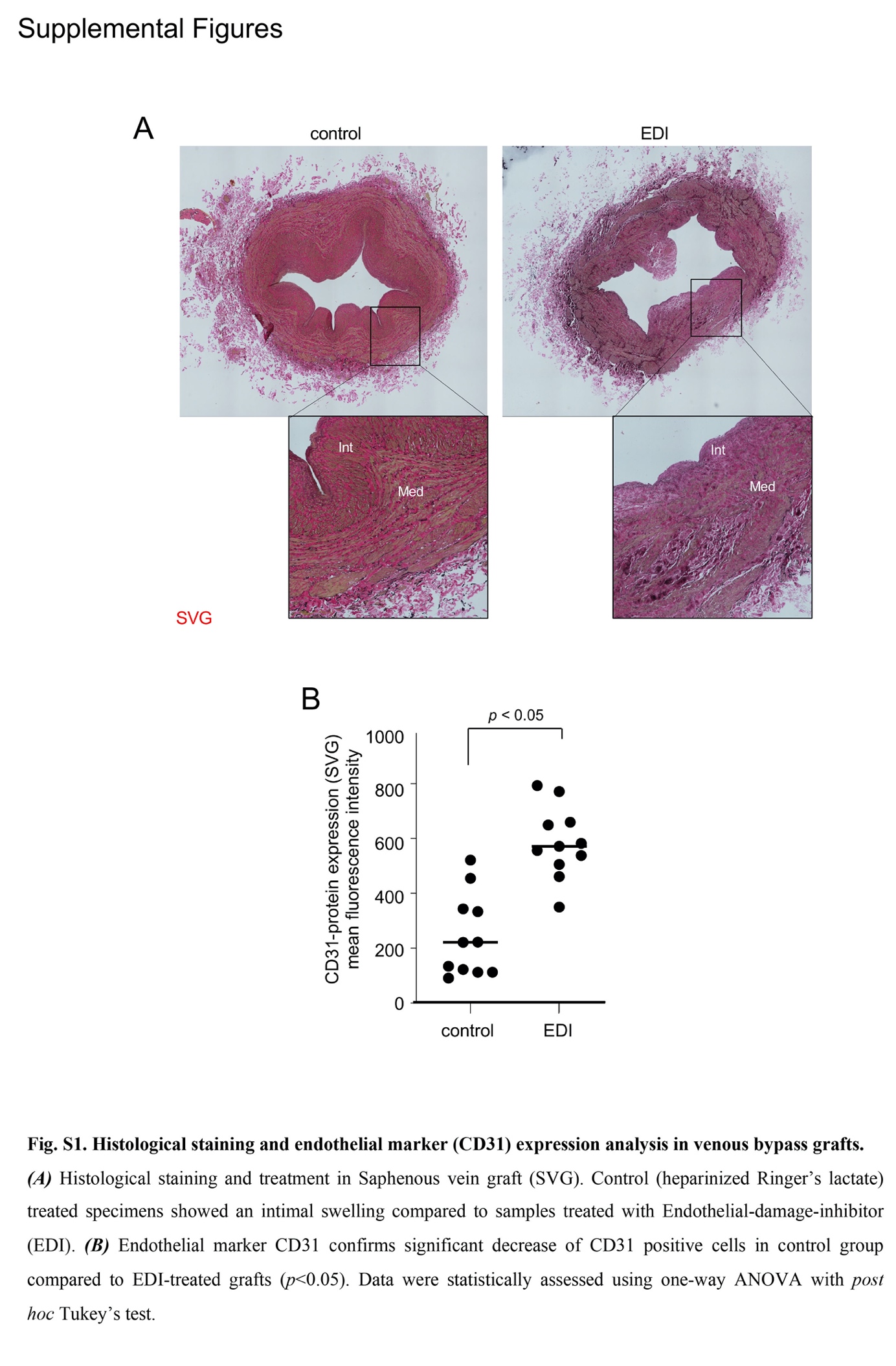
**

**
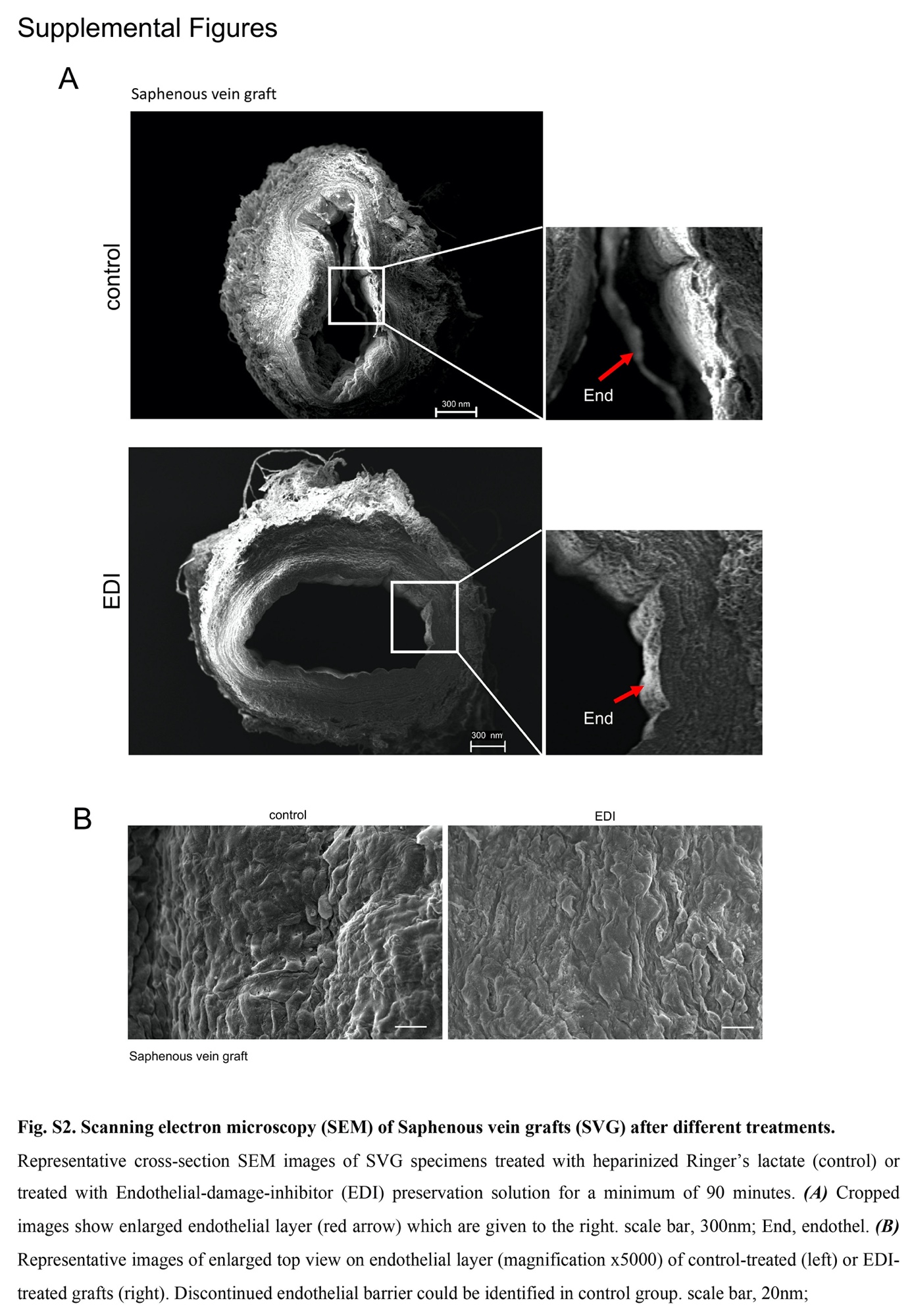

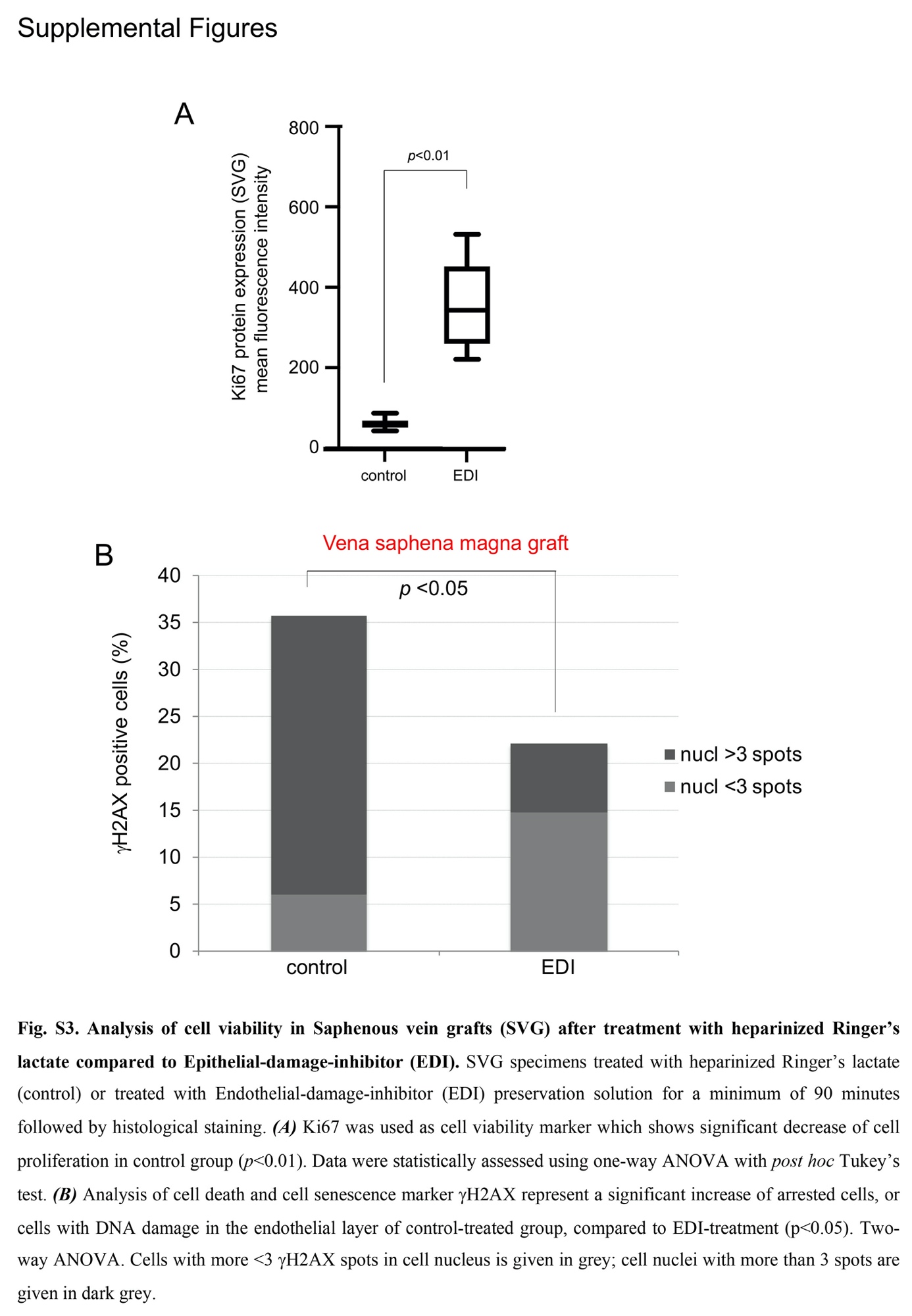
**

**
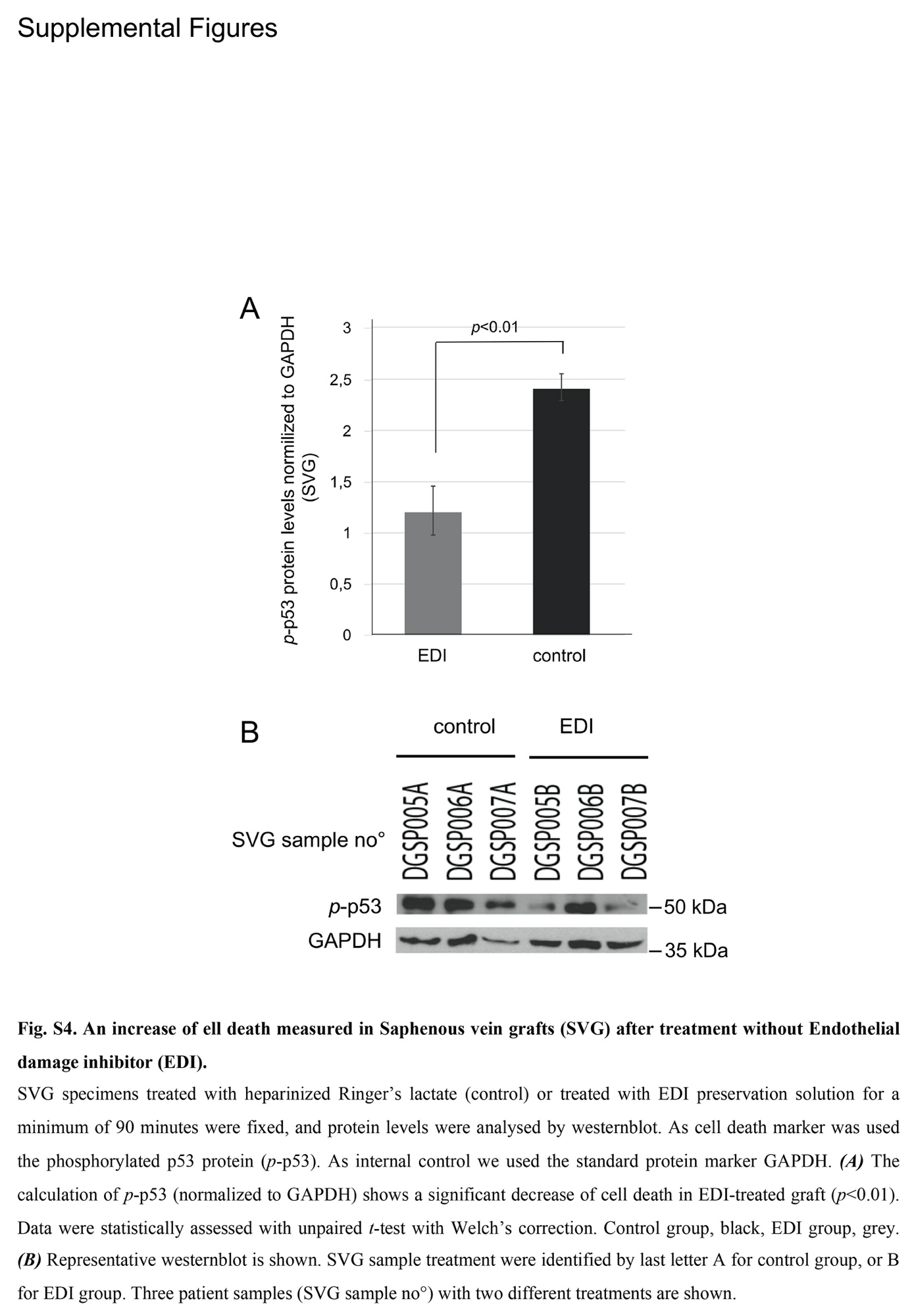
**

**
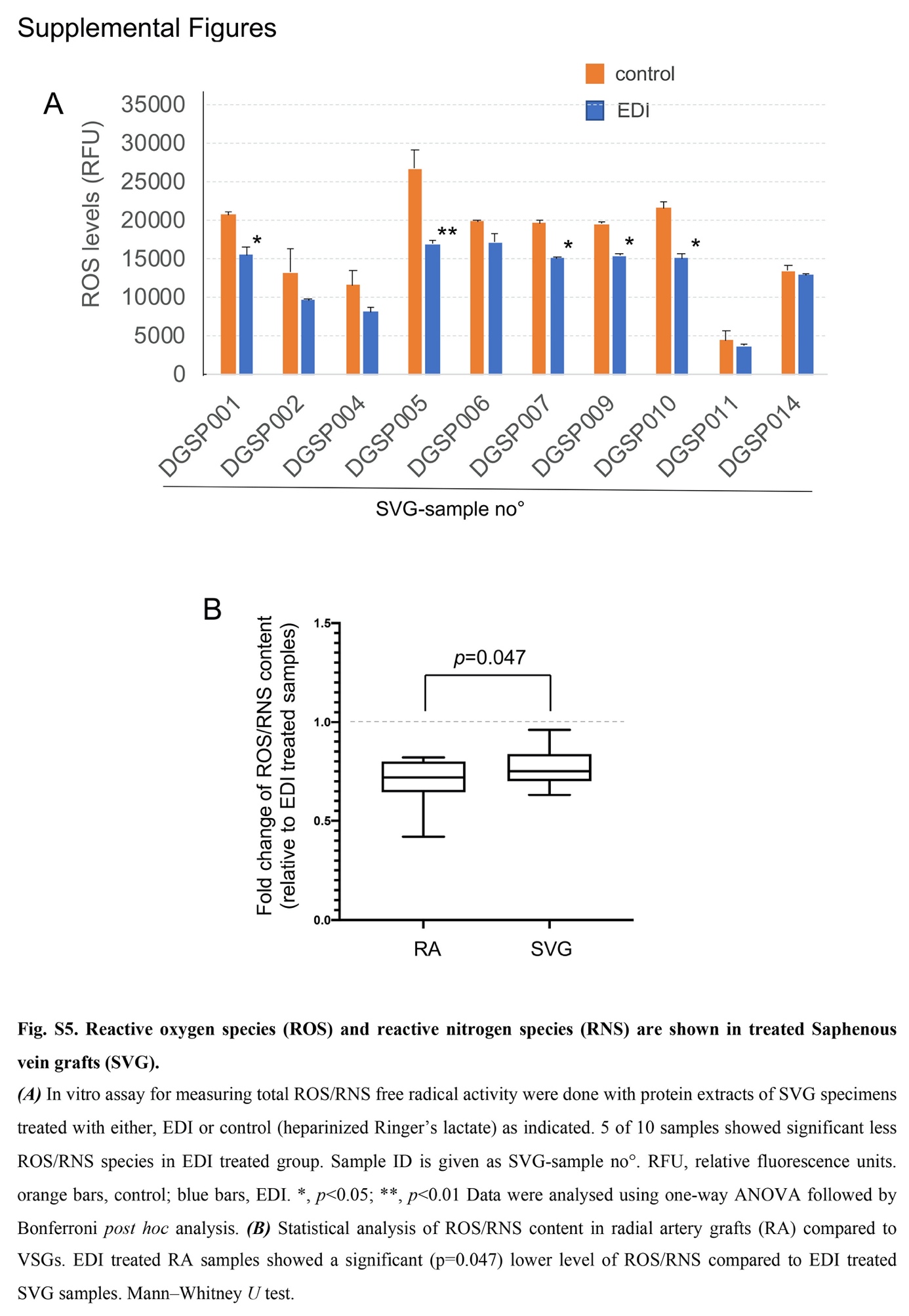
**

**
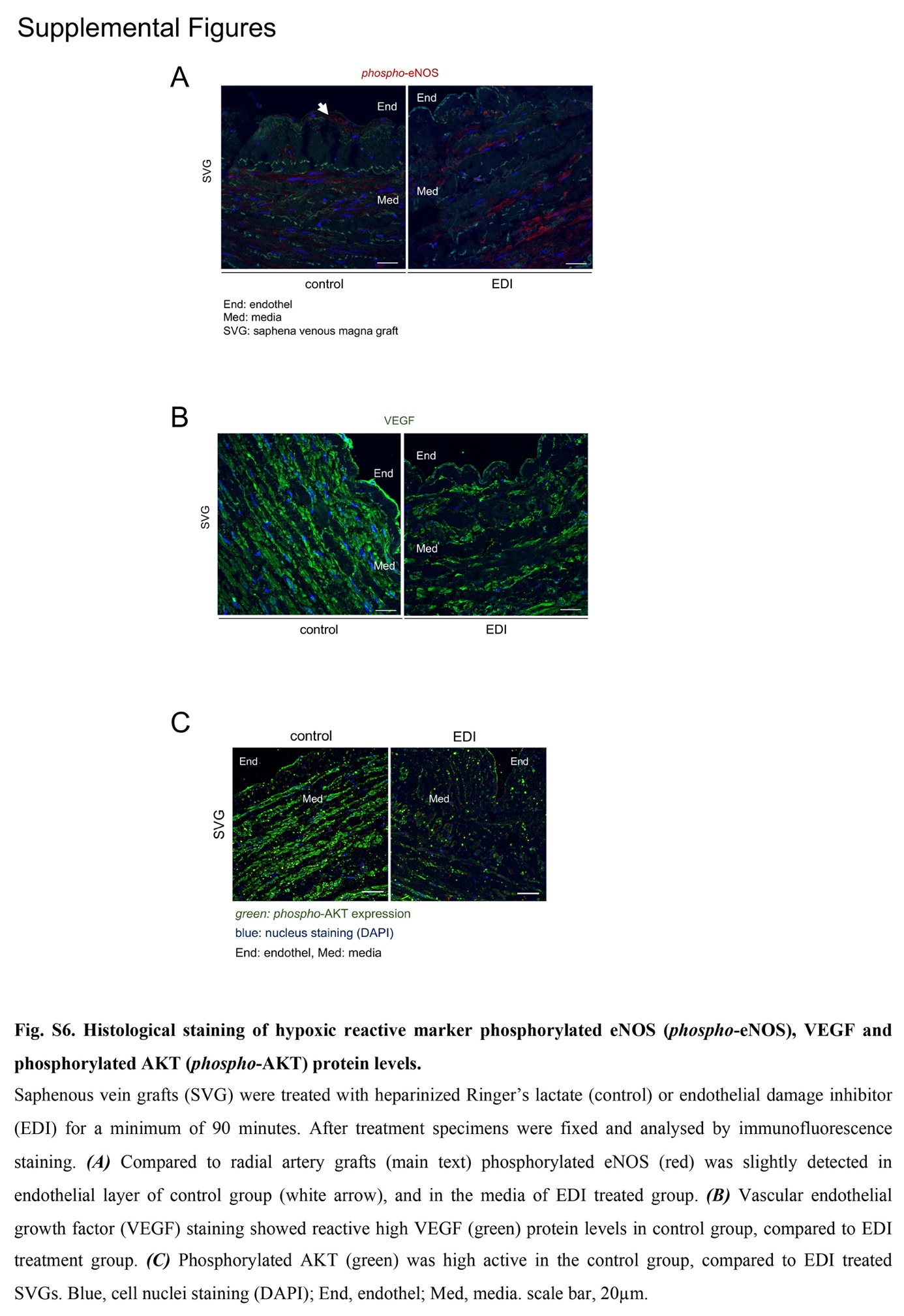
**

**
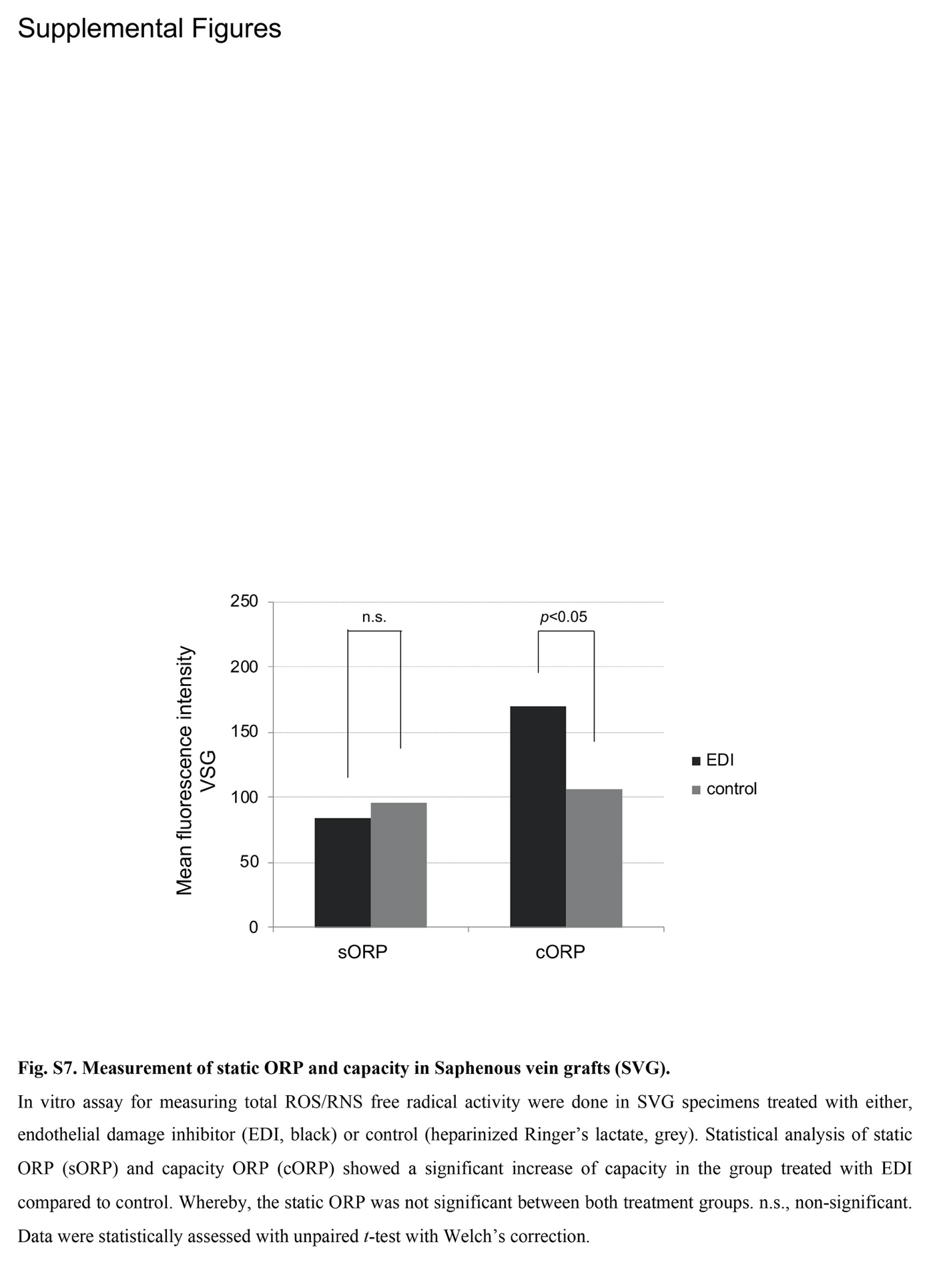
**

Supplemental Tables

| **Table I** | **Characteristics of the study population** |  |  |  |  |  |  |  |
| --- | --- | --- | --- | --- | --- | --- | --- | --- |
|  |  | **Study population** | | **RA grafts** | | **SVG grafts** | | ***P* Value** |
|  |  | **(*n* = 23)** | | **(*n* = 10)** | | **(*n* = 13)** | |  |
| Demographic, risk factors, and comorbidities | |  |  |  |  |  |  |  |
|  | Age (years) (range) | 66.2 | (48 - 80) | 62.7 | (48 - 75) | 68.7 | (56 - 80) | 0.08 |
|  | female, *n* (%) | 3 | (13.0) | 2 | (20.0) | 1 | (7.7) | 0.20 |
|  | Body mass index (BMI), *n* (%) | 28.8 | (20 - 42) | 30.6 | (22 - 42) | 29 | (20 - 42) | 0.46 |
|  | Adipositas (BMI >30), *n* (%) | 10 | (43.5) | 4 | (40) | 6 | (46.2) | 0.46 |
|  | Smoker, *n* (%) | 9 | (39.1) | 4 | (40) | 6 | (46.2) | 0.44 |
|  | Hypertension, *n* (%) | 22 | (95.7) | 10 | (100) | 12 | (92.3) | 0.17 |
|  | Dyslipidaemia, *n* (%) | 21 | (91.3) | 10 | (100) | 11 | (84.6) | 0.08 |
|  | Chronic renal failure, *n* (%) | 2 | (8.7) | 0 | (0) | 2 | (15.4) | 0.08 |
|  | Diabetes, *n* (%) | 10 | (43.5) | 3 | (30) | 7 | (53.9) | 0.12 |
|  | COPD, *n* (%) | 4 | (17.4) | 2 | (20) | 2 | (15.4) | 0.44 |
|  | Positive family history, *n* (%) | 4 | (17.4) | 3 | (30) | 1 | (7.7) | 0.10 |
|  | Ejection fraction (<50%), *n* (%) | 8 | (34.8) | 3 | (30) | 5 | (38.5) | 0.49 |
| Therapeutics | |  |  |  |  |  |  |  |
|  | Oral diabetes therapy, *n* (%) | 4 | (17.4) | 4 | (40) | 2 | (15.4) | 0.23 |
|  | Statins, *n* (%) | 22 | (95.6) | 10 | (100) | 12 | (92.3) | - |
|  | Aspirin, *n* (%) | 20 | (87.0) | 10 | (100) | 10 | (76.9) | **0.041** |
|  | ADP inhibitor, *n* (%) | 8 | (34.8) | 3 | (30) | 5 | (38.5) | 0.18 |
|  | NOAC, *n* (%) | 1 | (4.4) | 0 | (0) | 1 | (7.7) | 0.17 |
| Graft harvesting method | |  |  |  |  |  |  |  |
|  | Open, *n* (%) | 10 | (43.5) | 10 | (100) | 0 | (0) | - |
|  | Endoscopic, *n* (%) | 13 | (56.5) | 0 | (0) | 13 | (100) | - |
|  |  |  |  |  |  |  |  |  |
|  |  |  |  |  |  |  |  |  |
| COPD, chronic obstructive pulmonary disease; ADP, adenosine diphosphate; NOAC, novel oral anti-coagulant. | | | | | | | |  |
|  |  |  |  |  |  |  |  |  |

| **Table II** | **Correlation coefficients (ROS/RNS)** | |  |  |  |
| --- | --- | --- | --- | --- | --- |
|  |  | **RA grafts** | | **SVGs grafts** | |
|  |  | **Correlation coefficients (*r*)** | ***P V*alue** | **Correlation coefficients (*r*)** | ***P V*alue** |
| Age |  | 0.20 | **<0.001** | 0.25 | **<0.001** |
| Gender |  | 0.01 | n.s. | -0.02 | n.s. |
| Body mass index (BMI) | | -0.02 | n.s. | 0.03 | n.s. |
| Adipositas (BMI >30) | | 0.06 | n.s. | 0.01 | n.s. |
| Smoker |  | 0.12 | n.s. | 0.12 | n.s. |
| Hypertension |  | 0.09 | n.s. | 0.05 | n.s. |
| Dyslipidaemia |  | -0.03 | n.s. | 0.01 | n.s. |
|  | Statins | -0.06 | n.s. | 0.09 | n.s. |
| Chronic renal failure | | 0.01 | n.s. | 0.08 | n.s. |
| Diabetes |  | 0.23 | **<0.001** | 0.11 | n.s. |
|  | Oral diabetes therapy | 0.09 | n.s. | -0.04 | n.s. |
| COPD |  | 0.02 | n.s. | 0.02 | n.s. |
| Ejection fraction (<50%) | | -0.07 | n.s. | 0.05 | n.s. |
|  | Aspirin | 0.13 | n.s. | -0.1 | n.s. |
|  |  |  |  |  |  |
| COPD, chronic obstructive pulmonary disease. | | |  |  |  |

| **Table III** | **Correlation coefficients (eNOS)** | |  |  |  |
| --- | --- | --- | --- | --- | --- |
|  |  | **RA grafts** | | **SVGs grafts** | |
|  |  | **Correlation coefficients (*r*)** | ***P V*alue** | **Correlation coefficients (*r*)** | ***P V*alue** |
| Age |  | 0.23 | **<0.001** | 0.26 | **<0.001** |
| Gender |  | 0.01 | n.s. | 0.02 | n.s. |
| Body mass index (BMI) | | 0.05 | n.s. | -0.03 | n.s. |
| Adipositas (BMI >30) | | 0.02 | n.s. | 0.06 | n.s. |
| Smoker |  | 0.11 | n.s. | 0.02 | n.s. |
| Hypertension |  | -0.03 | n.s. | 0.07 | n.s. |
| Dyslipidaemia |  | 0.10 | n.s. | 0.04 | n.s. |
|  | Statins | 0.02 | n.s. | 0.11 | n.s. |
| Chronic renal failure | | -0.02 | n.s. | 0.02 | n.s. |
| Diabetes |  | 0.11 | n.s. | 0.12 | n.s. |
|  | Oral diabetes therapy | 0.04 | n.s. | -0.02 | n.s. |
| COPD |  | 0.03 | n.s. | 0.09 | n.s. |
| Ejection fraction (<50%) | | 0.12 | n.s. | 0.10 | n.s. |
|  | Aspirin | 0.14 | n.s. | -0.01 | n.s. |
|  |  |  |  |  |  |
| COPD, chronic obstructive pulmonary disease. | | |  |  |  |

**Supplemental Material and Methods**

***Antibodies used in this study***

Primary antibodies were γH2AX (Abcam, Anti-gamma H2A.X (S139 antibody [9F3], ab26350), or GAPDH (14C10) rabbit IgG mAb (Cell Signaling Technology, CST, 2118), Anti-CD31 (M0823, DAKO), TGFβ (CST, 37115), Anti-vWF (Abcam, ab6994), Anti-Ki67 (Abcam, ab15580), Anti-phospho-p53 (S15) (Abcam, ab1431), Anti-BP53 (Abcam, ab270645), Anti-VEGF (Abcam, ab1316), Anti-eNOS (CST, 9572), Anti-phospho-eNOS (Ser1177) (CST, 9571), Anti-phospho-AKT (Ser473) (CST, 0271), Anti-AKT (CST, 9272S), Anti-CAV-1 (Abcam, ab18199), Anti-HO-1 (Abcam, ab13243), Anti-PDGF-*a* (D1E1X) (CST, 3174S), and Anti-PDGF−β (28E1) (CST, 3169).

Purity of isolated cells was assessed using Western blot analyses with anti-CD90 antibody (D3V8A, Cell Signaling Technology) and anti-alpha vSMC actin antibody (Abcam, 4A4, ab119952).

Secondary antibodies: Goat anti-Rabbit IgG (H+L) Secondary Antibody, Alexa Fluor 546 or Goat anti-Mouse IgG (H+L) Secondary Antibody, Alexa Fluor 488 (molecular probes, ThermoFisher Scientific, Vienna, Austria).

***Oligonucleotide primer sequences used in this study***

vWF_fwd-AGGAAAGCCCATCAGACTAACAG; vWF_rev-GGCCTGGTGGTGACAAAATCT; GAPDH_f-TGCACCACCAACTGCTTAGC; GAPDH_r-GGCATGGACTGTGGTCATGAG; RPLP0_f-AGCCCAGAACACTGGTCTC; and RPLP0_r-ACTCAGGATTTCAATGGTGCC.
